# Synchronous Double Primary Cancer Complicated With Severe Hypercalcemia

**DOI:** 10.7759/cureus.44272

**Published:** 2023-08-28

**Authors:** Martim Alçada, Vasco Gaspar, Guilherme Cunha, José Pedro Manata, Filomena Roque

**Affiliations:** 1 Internal Medicine, Hospital Distrital Santarém, Santarém, PRT; 2 Internal Medicine, Hospital de Santarém EPE, Santarém, PRT; 3 Internal Medicine, Hospital Distrital de Santarém, Santarém, PRT

**Keywords:** malignant hypercalcemia, : prostate cancer, carcinoma of larynx, severe hypercalcemia, multiple primary cancer

## Abstract

Hypercalcemia of malignancy (HCM) is an important cancer-related medical emergency. It is a sign of advanced disease with a poor prognosis.

We report a case of a 55-year-old man who presented with decreased sensorium, constipation for 4 days, dysphonia, and weight loss for the past three months.

The physical examination showed a petrous nodular lesion of the neck in relation to the right sternocleidomastoid muscle. The digital rectal examination showed an enlarged prostate with a nodule of hard consistency. The blood revealed a hypercalcemia of 18.9 mg/dl and a prostate-specific antigen of 319.18 ng/ml. After further investigation, we discovered a squamous cell carcinoma of the larynx with multiple osteolytic bone lesions and a prostate adenocarcinoma.

The hypercalcemia was treated with sodium pamidronate with good results. Such severe hypercalcemia demanded further research which revealed that not only the osteolytic lesions contributed to the elevation of calcium serum levels but also the tumor secretion of parathyroid hormone-related protein.

This case highlights the importance of not only having a high suspicion for malignancy in patients presenting with hypercalcemia but also being aware of possible additional diagnoses in a patient with an already identified primary pathology.

## Introduction

The definition of multiple primary tumors (MPTs) is the existence of at least two either synchronous or metachronous cancers in an individual. The incidence of multiple primaries varies depending on the primary cancer type and what treatment was applied for these cancers. MPTs represent between 2.4% and 8%, up to 17% in a cancer population, as found in a study done over 20 years [1]. In a single-center retrospective study, with data retrieved from 109,054 cancer patients aged ≥18, that considered the Surveillance Epidemiology and End Results (SEER) definition of synchronous multiple primary cancers (less than a 2-month period), of the 1.63% of patients with MPTs only 29.13% were considered synchronous [2].

Aetiological factors that may predispose patients to multiple primaries can be grouped into host-related, lifestyle factors, and environmental influences. Caucasian ancestry, index cancer diagnosed at a younger age, lower stage and with generally indolent clinical behavior with longer survival, as well as positive family history are reported to harbor excess risk for multiple primaries [2].

Hypercalcemia is most commonly caused by malignancy and secondly by primary hyperparathyroidism. It can be a complication in 10-30% of all cancer patients. It is associated with poor prognosis since it is mostly found in advanced-stage cancer, the median duration of survival, for these patients, is of two to six months from disease onset. In an early-stage disease only 1-5% present with high serum calcium levels. The cancers that most usually present with hypercalcemia are multiple myeloma, followed by breast, renal, and squamous cell carcinomas [3].

Here, we present a case of a man who suffered from synchronous double primary cancer complicated with severe hypercalcemia.

This case report was previously presented as a poster at the 18th European Congress of Internal Medicine (ECIM) which took place from 29 to 31 August 2019.

## Case presentation

A 55-year-old male (with a Barthel index of 90) arrived at the emergency department presenting with unintentional weight loss of 10 kg (16% of total body weight) over a three-month period associated with anorexia, hoarseness, fatigue, altered sensorium, and constipation for four days. His past medical history included pulmonary and pleural tuberculosis treated 20 years prior and laryngeal tuberculosis treated five years prior. He stopped smoking 4 months before the episode with a tobacco exposure of 30 pack-year and consumed an average of 45 g of alcohol per day. His usual medication was a bronchodilator therapy consisting of aclidinium-formoterol twice daily.

On the physical examination, the most prominent finding was a nodular mass localized at the left cervical region in relation to the sternocleidomastoid muscle, of petrous consistency and non-movable. Also found an enlarged prostate gland with a hard consistency nodule at digital rectal examination. The remaining evaluation was unremarkable.

The blood tests showed a hypercalcemia of 18.9 mg/dl (corrected to albumin levels) and a prostate-specific antigen (PSA) of 319.18 ng/ml with no previous PSA elevations on record. Hemoglobin value of 14.1 g/dL, peripheral white cell blood count of 21.3 x10^9/L (neutrophils 15.7 x10^9/L, lymphocytes 15.7 x10^9 /L), and a platelet count of 309 x 10^9/L; urea 130.4 mg/dl, creatinine 1.8 mg/dl, and C-reactive protein 9.94 mg/dL.

Later results from blood tests also revealed a parathyroid hormone-related peptide (PTHrP) of 5.5 pmol/L (reference range <1.3).

**Figure 1:**
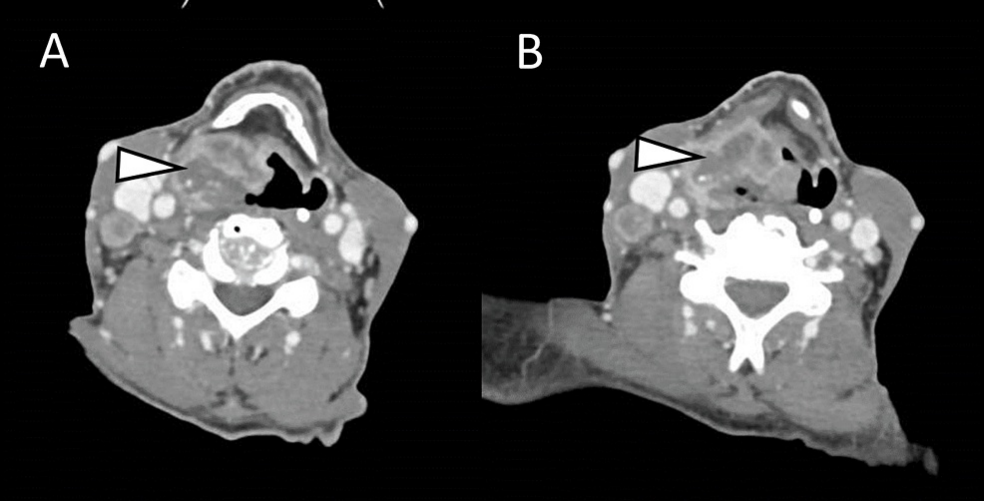
Contrast-enhanced computed tomography of the neck, thorax, abdomen, and pelvis Expansive infiltrative lesion localized at the right piriform recess and epiglottis, with a diameter of 4.5 cm and an ulcerated center. The lesion extends inferiorly to the right supraglottic fat tissue above the cricoid cartilage and the arytenoids, crossing over the midline. Arrowheads point to the laryngeal carcinoma.

Computer Tomography of the neck, thorax, abdomen, and pelvis showed an invasive, infiltrative lesion, localized at the piriform recess, with 4.5 cm of diameter and multiple osteolytic bone lesions with mostly axial distribution (Figure 1).

The patient was admitted to the hospital for further investigation of the infiltrative lesions and for the treatment of the hypercalcemia. He was given 90 mg of sodium pamidronate to treat the hypercalcemia with good results. During hospitalization, due to the osteolytic bone metastasis, the patient started experiencing pain mostly localized at the thoracic and lumbar regions, with increasing demand for analgesia with transdermal fentanyl and oral morphine for rescue treatment.

**Figure 2:**
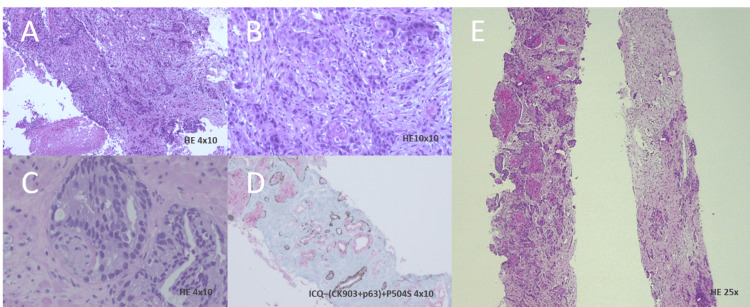
Tissue biopsy of the larynx, costal arch and prostate (A) and (B): Hematoxylin and eosin staining of larynx tissue fragments obtained by curettage at a magnification of 4x10 and 10x10, showing a squamous cell carcinoma well to moderately differentiated extensively ulcerated and with necrosis. (C) Hematoxylin and eosin staining of tissue from prostate biopsy at a magnification of 4x10, showing an acinar adenocarcinoma of the prostate with a Gleason score of 8. (D) Immunohistochemical studies with ICQ-(CK903+p63)+P504S at a 4×10 magnification of prostate tissue, showing PIN (prostatic intraepithelial neoplasia) lesion and associated high-grade lesion. (E) Hematoxylin and eosin staining of costal arch biopsy at a magnification of 25x, showing bone sequestering with 90% infiltrations by squamous cell carcinoma and partial necrosis (5%)

For histologic identification of the discovered lesions, a biopsy of the larynx, costal arch, and prostate tissue was taken revealing a squamous cell carcinoma of the larynx, bone tissue with 90% infiltration by a squamous cell carcinoma, and an acinar cell carcinoma of the prostate with a Gleason score of 8 (Figure 2).

**Figure 3:**
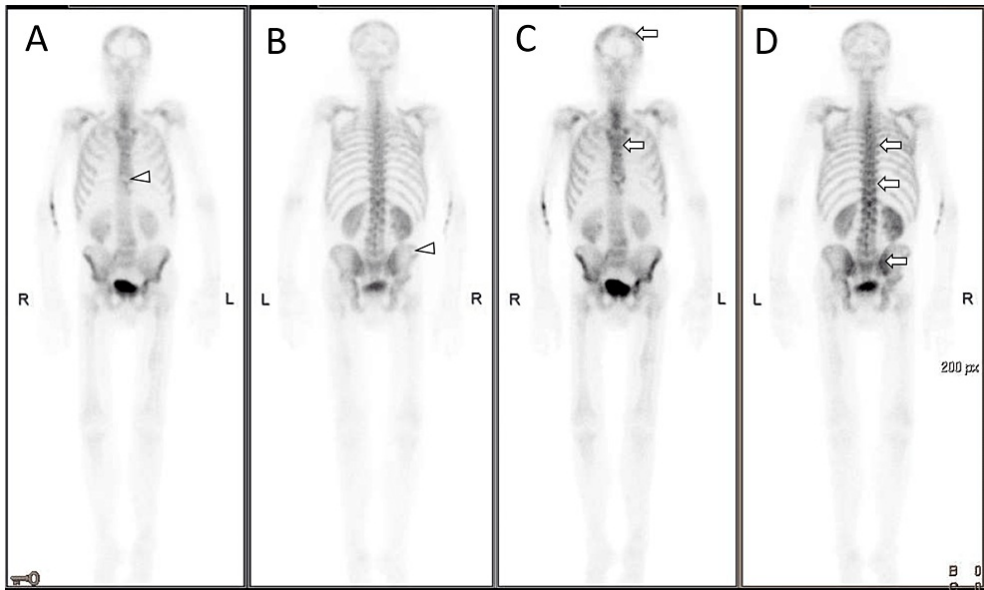
Bone scintigraphy Extensive bone metastasis with axial distribution, more pronounced at the skull, vertebral column, sternum, scapula, and hip bone (arrows). Osteolytic lesions can be seen at the distal 1/3 of the sternum and the superior portion of the iliac wings (arrowheads). (A) Anterior view scintigraphy acquired early after contrast. (B) Posterior view scintigraphy acquired early. (C) Anterior view scintigraphy acquired late. (D) Posterior view scintigraphy acquired late.

A bone scintigraphy revealed extensive bone metastasis with osteolytic lesions at the sternum and hip bone (Figure 3).

During hospitalization, the patient became increasingly dependent, reaching a Barthel score of 60. After the normalization of the serum calcium levels, reaching pain control, and completing the diagnostic investigation the patient was discharged. He was referred to be followed at a specialized cancer center for oncology and palliative medicine. After discharge the clinical status of the patient continued to worsen and he became increasingly dependent, which resulted in his returning to the hospital only 4 days later. The patient passed away on the fourth day of admission.

## Discussion

Synchronous MPTs are rarely found in daily clinical practice. The fact that the two cancers described in this case presentation were identified on the same day makes it even less frequent compared to other cancers that also fall in this category but were discovered over a larger time span. The complication with severe hypercalcemia in this case is also of notice, since from the aspect of its pathophysiology the multiple mechanisms involved make it very complex.

The most commonly used definitions for differentiating between synchronous and metachronous multiple primaries currently used are provided by the Surveillance, Epidemiology, and End Results (SEER) project and the International Association of Cancer Registries and International Agency for Research on Cancer (IACR/IARC). They differentiate from each other in regards to considering certain cancers separate or the same in regards to location, for example, the colon is regarded as one by the IACR but the SEER considers single tumors of different locations within the colon to be single primaries. There are also differences regarding the time gap limit for the primaries to be considered synchronous, as a two-month period is recommended by SEER whereas the IARC suggests an interval of less than 6 months. This results in significant differences by several percentage points in retrospective studies that do not use the same parameters in the selection and characterization of MPTs [1]. For example, a 25-year, single-center, retrospective study using the SEER definition, that included 1785 cancer patients, found that of this patient pool, only 1.63% had multiple primary cancers. Of the 1.63%, 70.87% were considered metachronous, which means that from the initial pool of 1785 cancer patients, only 0.47% had multiple synchronous primary cancers [2]. 

The patient in this case report presented with both a laryngeal squamous cell carcinoma and a prostate adenocarcinoma. Laryngeal cancers occur more commonly in men (5.8 cases per 100,000 vs. 1.2 per 100,000 in women). The 5-year survival rate has decreased over the past 40 years, from 66% to 63%, which is rare as most other cancers tend to have increased their survival rates [4]. Smokers are at 10 to 15 times higher risk of developing laryngeal cancer and heavy smokers can have up to 30 times increased risk. Alcohol as a risk factor is also well documented. The most common symptoms are hoarseness, dysphonia, dyspnea, and dysphagia [4]. The PTHrP is involved in laryngeal cancer cell growth and differentiation, existing in the isoforms 139, 141, and 173 amino acids. There is both in vivo and in vitro documented evidence of PTHrP involvement in tumor growth, spread, and its beginning. Poorly differentiated oral squamous cell carcinomas and poor outcomes are connected to PTHrP overexpression [5].

In males, the second most frequent malignancy is prostate cancer (after lung cancer). In 2018 there were 1,276,106 new cases of which 358,989 died accounting for 3.8% of cancer-related deaths in men. There is a correlation between age and the incidence and mortality of prostate cancer, the average diagnosis age is 66 years. It can be asymptomatic at the early stage and usually has an indolent course, mostly requiring minimal or no treatment. Symptoms are similar to prostatic hypertrophy, those being frequency, nocturia, and difficulty with urination, but in later stages can present with retention and back pain due to metastasis to the axial skeleton being the most frequent location [6].

Etiology and pathophysiology

Hypercalcemia of malignancy is most commonly caused by increased bone resorption with the release of calcium from bone and the inadequate ability of the kidneys to manage higher calcium levels. There are four mechanisms: 1) Humoral hypercalcemia 2) Osteolytic hypercalcemia 3) Extra-renal production of 1,25-dihydroxyvitamin D, and 4) Ectopic parathyroid hormone (PTH) production [3].

The most common mechanism is humoral hypercalcemia via the secretion of PTHrP accounting for 80% of cancer-associated hypercalcemia. It can be associated with cancers with little or no bone metastasis. PTHrP binds to the same receptor PTH does (PTH1R), and so it mimics the PTH effect by activating similar pathways such as protein kinase A and C, cyclic adenosine monophosphate (cAMP), phospholipase C, and inositol phosphate. The cancers associated with this mechanism are squamous cell carcinomas (head, lung and neck, esophageal, cervical, and colon cancers), breast, kidney, bladder, endometrial, and ovarian cancers. Less frequently associated but also described are pancreatic neuroendocrine tumors, Hodgkin and non-Hodgkin lymphomas [3].

The second most common mechanism is osteolytic metastasis releasing large amounts of calcium from the bone and is responsible for 20% of cancer hypercalcemias. This is most commonly observed in multiple myeloma and metastatic breast cancer followed by leukemia and lymphoma. In this pathophysiologic mechanism, there is also the release of other humoral factors responsible for hypercalcemia [3].

In 1% of cases, the cause can be the excess production of extra-renal 1,25-dihydroxyvitamin D. This occurs nearly exclusively in lymphomas (both Hodgkin and non-Hodgkin) but is also described to happen rarely in ovarian dysgerminoma [3].

Finally, the last mechanism happens in around 1% of these cases and is due to the ectopic secretions of PTH by malignant cells. It has been found mainly in lung cancer, ovary, sarcomas, and neuroendocrine tumors [3].

When managing hypercalcemia what determines the timing and type of treatment used is the serum calcium levels and the associated symptoms. As such, severe hypercalcemia associated with symptoms must be treated immediately and the therapy should be aimed at the mediating mechanisms. Treatment includes volume overload, calcitonin, bisphosphonates, denosumab, corticosteroids, and cinacalcet. In patients with renal insufficiency or severe refractory hypercalcemia, hemodialysis should be considered [3].

With these mechanisms in mind, we can most likely assume that in the described case the two first mechanisms were responsible for the severe hypercalcemia found at admittance. There is evidence both of extensive osteolytic infiltrative lesions of multiple bones with mainly axial distribution and of high values of PTHrP. The values of PTH were normal, so the possibility of ectopic production of PTH by malignant cells and concomitant hyperparathyroidism was also ruled out.

## Conclusions

This case highlights a rare occurrence of multiple synchronous primary cancers, more specifically of the larynx and prostate. Both described mechanisms of hypercalcemia in malignancy were likely linked to the larynx cancer as its association with the secretion of PTHrP is well documented in the literature, unlike prostate cancer. Histologically there was clear extensive infiltration of bone by the laryngeal squamous cell carcinoma, likely contributing to the excessive release of calcium from the bone into the bloodstream.

Although the prostate adenocarcinoma spreads preferentially to the axial skeleton, the histology of the bone biopsy did not show any cells arising from the prostate malignancy.

Despite being able to control the initial complications associated with the malignancy, the outcome was still poor as predicted by the overexpression of PTHrP.
